# The Mediational Role of Self-Support Personality in the Association of Family Function and School Belonging in Adolescents

**DOI:** 10.3389/fpsyg.2021.790700

**Published:** 2022-01-04

**Authors:** Zhendong Yao, Lu Pang, Jin Xie, Wei Xiang, Huiying Yu, Wei Hu

**Affiliations:** ^1^School of Public Administration, Hunan Normal University, Changsha, China; ^2^School of Preschool Education, Hunan College for Preschool Education, Changde, China; ^3^Mental Health Service Center, Huanghuai University, Zhumadian, China; ^4^Mental Health Service Center, Changsha University, Changsha, China; ^5^School of Marxism, Guizhou Medical University, Guiyang, China

**Keywords:** family function, school belonging, adolescents, self-support, personality

## Abstract

Some previous studies have explored the impact of family function on school belonging. However, little is known about the parallel mediating relationship underlying them. This study aims to investigate the formation mechanism of school beginning in a sample of Chinese adolescents and examined the parallel mediating role of interpersonal self-support and individual self-support in the link between family function and school belonging. A cross-sectional study was conducted in four schools of the district of Hunan province in China, and 741 students were surveyed using cluster sampling. Family cohesion and adaptability scale (*FACES*), Adolescent students self-supporting personality scale (*SSPS-AS*), School belonging scale were applied. The results indicated that interpersonal self-support and individual self-support, together, and uniquely, parallel mediated the relationship between family function and school belonging. It can be concluded that family function not only has direct effects on school belonging but also has indirect effects through interpersonal self-support and individual self-support.

## Introduction

Sense of school belonging refers to students’ feeling that they are liked, respected, and valued by others in the school (Hamm and Faircloth, 2005), which has been received great attention in social life and empirical research (Ahmadi et al., 2020). Previous research revealed its great effect on individual development, especially in adolescents. For example, Dixson and Scalcucci (2021) found that school belonging improved the decision-making, motivation, and self-management of adolescents. Wormington et al. (2016) found that school belonging reduced adolescent peer injury. It is necessary to explore the mechanism of improving students’ sense of school belonging. Furthermore, the previous study has highlighted the importance of research focusing on the sense of school belonging in Eastern Cultures because of its poor performance comparing to the Western Cultures (Cortina et al., 2017). In order to achieve the attachment needed for contentment and learning, a feeling of ‘belonging’ must develop (Allen and Boyle, 2018). Therefore, this study based on the theory of family and peer systems linkage examined the association of family function and school belongings with Chinese adolescents, and further explored the mediating effect of self-support personality.

### Family Function and School Belonging

Adolescence is a “stormy and rainy period” of physical and psychological development, and their family is the main area for everyday life. Family is a microsystem, which is the environment that individuals are in and have a direct connection with themselves (Bronfenbrenner, 1979; Belsky, 1981). The family system theory puts forward from the macro level, the function of the entire family system has an important influence on the growth process of the child. The better the family function, the healthier the child will be physically and mentally (Beavers and Hampson, 2002). Although the structure and composition of the family have changed significantly in the past decades, the family is still the main source of everyone’s physical, emotional, and social support (Palmer and Glass, 2003). For adolescents, the support system provided by the family is particularly important (Steinberg, 2010). On the contrary, family dysfunction may be accompanied by more family contradictions and conflicts, leading to internalization problems such as anxiety and depression, or externalization problems such as aggression and substance abuse (Soloski et al., 2016).

Family function refers to the emotional connection, family rules, family communication, and the effectiveness of dealing with external events of members in the family system. The study mainly selects two aspects of family function, including family cohesion and family adaptability. Family cohesion is defined as the emotional bond between family members (Olson, 2000). Family adaptability refers to the ability of the family system to change with the family situation and problems in different stages of family development (Wu et al., 2019). By analyzing the relationship between family cohesion and school belonging, the following findings were obtained. Family cohesion is a good predictor of adolescent mental health (Fosco and Lydon-Staley, 2020) and has an important protective effect against internalizing problems, such as depression and anxiety (Shah et al., 2021). Family cohesion promotes individuals to form a positive mood in the short term and to promote individuals to form long-term resilience and well-being (Fosco and Lydon-Staley, 2019). On the contrary, adolescents with low family cohesion are more likely to hold a negative attitude toward themselves, encounter more difficulties in school life and show more externalized behavior problems (Liu et al., 2014). It can be seen that family cohesion is a powerful guarantee for cultivating healthy individuals and for students to better integrate into the school. At the same time, previous studies have proved that family cohesion can significantly and positively predict school belonging (Qin et al., 2015).

By analyzing the relationship between family adaptability and school belonging, the following findings were obtained. Family adaptability has a positive effect on individual development; for example, highly adoptive families are biased toward high creativity scores (Gardner and Moran, 1990). Family adaptability has a protective effect on adolescent mental health, for example, there is a negative relationship between adolescent family adaptability and depression (Li et al., 2021). Positive personal characteristics emerged as the strongest predictors of school belonging when family adaptability promoted individual development (Allen et al., 2018). Family adaptability reflects the adaptation of families to environmental changes, while family adaptation to the environment promotes the development of individual security. Because the realization of family function provides certain environmental conditions for the healthy development of physical, psychological, and social aspects of family members (Oliveira et al., 2014). Moreover, there is a relationship between security and school belonging (Slaten et al., 2016). Previous studies have pointed out that students’ adaptation to schools is related to family adaptability (Cho, 2010). The above findings were obtained by analyzing the relationship between family adaptability and school belonging.

### Self-Support as a Mechanism Through Which Family Function to School Belonging

Self-support personality is a Chinese indigenous personality construct (Xia et al., 2013b), an ideal personality emphasized by Chinese culture. Its main characteristics embody Confucius’ doctrine of the mean, collectivism, and Chinese culture of interdependence (Xia et al., 2013a). Self-support personality includes two dimensions, namely individual and interpersonal aspects; each aspect consist of personal independence, personal initiative, personal responsibility, personal flexibility, and personal openness (Xia et al., 2014b). The essence of personality effect results from interaction between personality and social environment (Barber, 1992). The development of personality depends on people’s social environment (Xia et al., 2014a). Environmental factors were important not only as interactional effects, as noted earlier, but also as significant main effects (Brook et al., 2001).

According to the interpersonal situational influence model, environmental factors will use the self-system as a mediator to affect the individual’s behavioral performance and ultimately lead to different developmental outcomes (Connell and Aber, 1994). Family is the basic unit of society, which is generally considered as the microsystem that has the greatest influence on individuals. The ability of the whole family to maintain positive and effective communication is regarded as an important part of social and personal skills (Connolly and O’Moore, 2003). The focus of cohesion construction is how the family system balances the separation and unity of its members (Olson, 2000). Adolescents from cohesive families showed more life satisfaction and social inclusion, while adolescents from divorced families showed more aggression and hostility (Qin et al., 2015). The higher the degree of family members’ love for each other, the higher the individual’s subjective satisfaction with family functions and the more the individual can solve the basic personal life problems encountered by themselves (Yang et al., 2014). It can be seen that family function has an important impact on the individual’s psychology.

Every child’s personality can choose the subjective psychological environment from the objective environment. This subjective psychological environment constitutes the background of individual personality development in the future (Huang et al., 2006). Part of the individual’s subjective psychological feelings come from the family. Family members who live with each other form a collective, and mutual support between members is conducive to developing an individual’s self-supporting personality. It has been pointed out that interdependence is an implication of the concept of self-support (Xia and Huang, 2006a,b); positive relationship schema may be the main foundation of self-supporting personality (Xia et al., 2012b).

Previous studies have carried out some similar explorations on the relationship between self-support personality and school belonging. Taking personality traits as a whole, it is concluded that there is a significant positive correlation between personality traits and school belonging. The specific analysis is as follow (Pang, 2009). People with high self-support have a more positive evaluation of themselves and others than those with low self-supporting (Xia and Geng, 2012). A school is a place where teachers and students live together. Adolescents’ positive self-evaluation helps individuals gain trust, be welcomed, and gain friendship in the collective. People who have stronger personal independence or initiative than others have positive self and another schema (Xia et al., 2013b). Self-supporting individuals are characterized by initiative (Xia and Huang, 2006b). Teenagers have many tasks to complete in school, and initiative is conducive to adolescents to better adapt to school life, such as actively participating in school-organized activities and actively integrating into collective life.

The experience of self-affirming, self-disclosing friendships holds the potential for adolescents to develop a sense of importance, trust, acceptance, and being understood within the school, key underpinnings of a sense of belonging (Hamm and Faircloth, 2005). So, as an important part of the self-system, does personality trait mediate between family function and school belonging? It can be seen that personality is related to individual development.

### The Present Study

This study tested the mediating role of self-support personality between adolescents’ family function and school belonging. In the study, the two dimensions of self-support personality, interpersonal self-support and individual self-support, are put into the model for analysis. Then, it compares the role of interpersonal self-support and individual self-support in adolescents’ family function and school belonging. Based on previous literature analysis, we proposed the following assumptions:

Hypothesis 1. Interpersonal self-support and individual self-support act as parallel mediate role between adolescents’ family function and school belonging.

Hypothesis 2. The effect value of the parallel intermediary between interpersonal self-support and individual self-support in adolescents’ family function and school belonging is different.

## Materials and Methods

### Participants and Procedures

Participants were selected from five junior high schools in Hunan Province by random sampling, China. Before carrying out the online questionnaire survey, all examiners were trained to inform them of the precautions, and then each examiner was stationed in the corresponding classes of each school. After obtaining the consent of the subjects, the examiners told the subjects how to fill in the online questionnaire. Then, the subjects voluntarily fill in the online questionnaire, click the “submit” button after filling in, and the corresponding data can be collected in the background.

A total of 780 questionnaires were distributed, and 741 valid questionnaires were obtained, with an effective rate of 87.18%. All the respondents were junior high school students, among whom 328 were boys, accounting for 44.26%, 413 were girls, accounting for 55.74%, 246 were in grade one, accounting for 33.20%, 311 were in grade two, accounting for 41.97%, 184 were in grade three, accounting for 24.83%. Their mean age was 13.82 years (*SD* = 0.91, range 12–16).

### Measures

#### Family Cohesion and Adaptability Scale

The second edition of the family cohesion and adaptability scale (FACES II) was compiled by Olson et al. (1982). The revised family function scale was used in the current study. The scale includes 30 items, which are divided into two dimensions: cohesion and adaptability. Among them, there are 16 items of cohesion, such as “in our family, entertainment activities are done by the whole family together,” and 14 items of adaptability, such as “when there is a conflict in the family, members compromise with each other.” Five points are used to score, 1 means “no,” 2 means “occasionally,” 3 means “sometimes,” 4 means “often,” 5 means “always.” The higher the score, the better the family adaptability and cohesion. The revised scale has good reliability and validity (Wang et al., 1999). In this study, the Cronbach’s alpha of the whole scale was 0.97.

#### Adolescent Students Self-Supporting Personality Scale

The self-support personality scale of young students was compiled by Xia et al. (2008). The scale includes two subscales, namely, the individual self-support scale and interpersonal self-support scale, each with 20 items (Xia and Huang, 2008). For example, the items of personal independence “like to arrange what needs to be done in advance” (positive scoring); the items of interpersonal independence “usually dare not go to other people’s home alone” (reverse scoring). The scale has a 5-point score, and the higher the score, the higher the level of related traits (Xia and Huang, 2009). The scores of sub-dimensions are obtained by calculating the total scores of the items corresponding to each sub-dimension. In this study, the Cronbach’s alpha of the whole scale was 0.87.

#### Psychological Sense of School Membership Scale

At first, it was compiled by Goodenow, and then Pan et al. (2011) revised the school belonging scale to form a Chinese version. There are 18 items on the scale, which are divided into three dimensions: a sense of belonging, identity, and school attachment. The scale has good reliability and validity. Among them, there are 7 items of sense of belonging, such as “the students in this school seriously adopt my opinions”; 8 items of identity, such as “the people in this school are very friendly to me”; 3 items of school attachment, such as “in this school, I can be myself.” Using 5 points, 1 means “never like this,” 2 means “slightly not like this,” 3 means “ordinary,” 4 means “slightly like this,” 5 means “always like this”(Pan et al., 2011). In this study, the Cronbach’s alpha of the whole scale was 0.79.

### Data Analysis

In this study, SPSS 26.0 and AMOS 26.0 were used to manage and analyze the data. Among them, descriptive statistics completed by SPSS 26.0 and model fitting and path analysis were completed by AMOS 26.0. The data was analyzed before the formal test. According to Harman’s single factor test, the common method deviation was tested. The results showed that there were 21 factors whose eigenvalues were greater than 1. The explained variance percentage of the first factor was 23.20%, which was far lower the 40% recommended by Podsakoff et al. (2003). This showed that there was no serious common method deviation in this study.

## Results

### Preliminary Analysis

The results of descriptive statistics and correlational analysis for the relationships between family function, self-support, and school belonging of junior high school students are presented in Table 1. The Family function was moderately and significantly related to school belonging (*r* = 0.64, *p* < 0.001). Meanwhile, family function was positively and significantly related to interpersonal self-support (*r* = 0.49, *p* < 0.001) and individual self-support (*r* = 0.42, *p*<0.001). Interpersonal self-support (*r* = 0.49, *p* < 0.001) and individual self-support (*r* = 0.42, *p*<0.001) were positively and significantly related to school belonging.

**TABLE 1:** Descriptive statistics and correlation analysis for the main variable.

	M ± SD	1	2	3	4
1 Family function	3.94 ± 0.71	1			
2 Interpersonal Self-support	1.78 ± 0.25	0.49**	1		
3 Individual Self-support	3.45 ± 0.40	0.42**	0.52**	1	
4 School belonging	3.71 ± 0.64	0.64**	0.49**	0.41**	1

*P < 0.05.

### Measurement Model

Before measuring the mediation effect model, we first test the measurement model, which included two latent constructs (family function, school belonging) and two observed variables (individual self-support and interpersonal self-support). The initial model measurement results was not well fitted: χ^2^ = 48.77, df = 10, *p* < 0.001, CMIN/DF = 4.88, CFI = 0.99, GFI = 0.98, RMSEA = 0.072. According to the modification indices, the two error terms of sense of school belonging were allowed to be correlated. One error term of sense of school belonging and one error term of family function were allowed to be correlated. According to the suggestion of model modification, the results were improved after the modification of the above model, which conforms to the corresponding measurement standards: χ^2^ = 15.72, df = 8, *p* < 0.05, CMIN/DF = 1.97, CFI = 0.99, GFI = 0.99, RMSEA = 0.036. Subsequently, we examined the proposed structural relationships in which family function was related to school belonging via interpersonal self-support and individual self-support under the control of control variable (gender and grade). The modified model indicated the following fit indices: χ^2^ = 60.25, df = 21, *p* < 0.00, CMIN/DF = 2.87, CFI = 0.99, GFI = 0.98, RMSEA = 0.050. The model also showed good model indices.

### Mediating Variables

**FIGURE 1:**
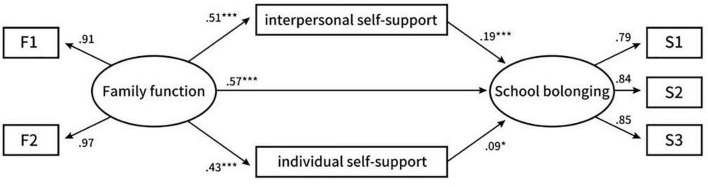
Standardized path coefficients for the proposed model. *P< 0.05, **P< 0.01, ***P< 0.001.

As shown in Figure 1, the standardized direct effect results show that there is a significant positive correlation between family function and interpersonal self-support (β = 0.38, *P* < 0.001), personal self-support (β = 0.31, *P* < 0.001) and school belonging (β = 0.036, *P* < 0.001). Interpersonal self-support is positively correlated with school belonging (β = 0.0030, *P* < 0.001), and individual self-support is positively correlated with school belonging (β = 0.04, *P* < 0.05). As shown in Table 2. The mediating effect of interpersonal self-support on family function and school belonging was significant (estimate = 0.088, SE = 0.019, 95% CI [0.054, 0.13]), and the mediating effect of individual self-support on family function and school belonging was significant (estimate = 0.034, SE = 0.015, 95% CI [0.0070, 0.0010]). The results showed that interpersonal self-support had a more significant mediating effect than individual self-support (beta = 0.054, SE = 0.026, CI [0.0040, 0.11]).

**TABLE 2:** Bootstrapping indirect effects and 95% confidence intervals (CI) for the model.

	Estimate	SE	95% CI	
			Lower	Upper
Family function → Interpersonal self-support → School belonging	0.094**	0.019	0.060	0.13
Family function → Individual self-support → School belonging	0.035**	0.015	0.0070	0.068
Interpersonal self-support—Individual self-support	0.059**	0.026	0.0090	0.11

**P < 0.01.

### Gender Difference

The results of the study measuring differences in the four latent variables by gender showed no gender differences in family functioning [*t*_(741)_ = 3.20, *p* = 0.095], interpersonal self-support [*t*_(741)_ = 0.19, *p* = 1.00], individual self-support [*t*_(741)_ = 1.80, *p* = 0.65], and school belonging [*t*_(741)_ = 2.45, *p* = 0.30]. Based on this, the model was further tested for gender differences.

Through the multi-group analysis function of AMOS 26.0, the differences of path coefficients between male and female models were tested. In the study, it is assumed that the two models grouped according to gender remain unchanged in Basic Parameters (Factor Loading, Error Variables, and Structural Covenants). One allowed free estimations of transgender paths (unconstrained structural paths). While the other limited the equality of path coefficients between two genders (constrained structural paths). The results showed that there is a significant difference between the two models [^△^χ^2^ (24, *N* = 741) = 58.89, *P* < 0.001] (see Table 3). At the same time, comparing other parameters between the two models, the research results showed that the indicators all meet the relevant requirements.

**TABLE 3:** Unconstrained and constrained structural paths across genders.

	χ2	df	CFI	RMSEA	SRMR	AIC	ECVI
Unconstrained	58.90	24	0.99	0.044	0.027	122.89	0.17
Constrained	87.10	38	0.99	0.042	0.043	123.07	0.17

Since χ^2^ is easily affected by large sample size and reaches a significant level, in order to verify the stability of the model’s transgender, we use the critical ratios of differences (CRD) of the two models as an indicator to further investigate the span of the structural model. Gender stability According to decision-making rules, the absolute value of CRD greater than 1.96 indicates that there is a significant difference between the two parameters (Arbuckle, 2003). The results showed that there is no significant difference between the variable structure paths (CRD[family function → chool belonging] = 0.11), CRD[family function → interpersonal self-support] = −0.71, CRD[family function → individual self-support] = 0.74, CRD[interpersonal self-support → school belonging] = −0.94, CRD[individual self-support → school belonging] = −0.20). The research showed no significant difference between the two models in transgender comparison; that is, the model has transgender stability.

## Discussion

The purpose of the present study was to examine the relationship between family function and school belonging through the potential mediation of interpersonal self-support and individual self-support. The modified structural equation modeling result fits the indices. Moreover, the findings of the path analysis showed that the parallel two-mediator model was established.

The results showed that family function has a gratitude significant direct effect on school belonging, interpersonal self-support, and individual self-support. These results were similar to these of previous studies and theories. Family provides people with a sense of belonging in their lives (Goodman et al., 2019). There is a positive correlation between family communication and adolescents’ use of strategies (Wang et al., 2002). Family cohesion can directly affect school belonging (Zhao et al., 2017). The good family function provides a warm and supportive environment for individuals, makes individuals feel more positive emotions, and show better adaptability in school (Mou et al., 2016). The results of this study showed that the better the family functioning of junior high school students, the better the corresponding sense of school belonging.

### Mediating Effect of Interpersonal Self-Support on the Association Between Family Function and School Belonging

The results showed that the family function predicts interpersonal self-support of junior high school students. The results of the research were similar to previous research and theories. The basic function of the family is to provide certain environmental conditions for the healthy development of physical, psychological, and social aspects of family members (Skinner et al., 2010). As the manager of children’s peer relationships, parents supervise and guide children’s activities every day. Family communication is considered as a means to generate cohesion and flexibility in the family, and good family function helps individuals form interpersonal personality quality. In contrast, unbalanced cohesion and flexibility are inevitably related to poor communication (Olson, 2000).

The results suggested that interpersonal self-support can predict school belonging. The results of the study were consistent with previous results and theories. Those with high interpersonal self-support had positive processing of interpersonal information, while those with low interpersonal self-support had negative processing of interpersonal information (Xia et al., 2013c). The peer relationship that children developed in early school may have the effect of promoting adaptive development (Chen et al., 2012). On campus, everyone often communicates and transmits information. The acquisition of interpersonal self-support helps junior high school students to form positive coping styles, such as actively collecting and processing information. In contrast, positive ways help junior high school students to accept others and be accepted by others. The growth experience of junior high school students will not be smooth sailing, and may encounter difficulties and setbacks in the growth process. Existing studies have pointed out that interpersonal self-support is protective for the growth of individuals. For example, interpersonal self-support characteristics indicate the decline of aggressiveness (Fan et al., 2018). Moreover, interpersonal responsibility can be used as a personality protection factor for depression (Xia et al., 2012a), interpersonal independence is a protective personality factor for depression (Xia et al., 2011b), the self-schema and others’ schema of high self-support are more active than those of low self-support (Xia and Geng, 2012). When a family encounters a crisis, the stronger the ability to use its internal and external resources to solve problems, the more the individual can solve the basic interpersonal problems encountered by themselves (Yang et al., 2014). According to the above analysis, interpersonal self-support is established as a mediator variable between family function and school belonging.

### Mediating Effect of Individual Self-Support on the Association Between Family Function and School Belonging

The study showed that individual self-support mediated the relationship between family function and school belonging, suggesting that family function increase junior high school students’ self-support which in turn increases their school belonging. Healthy families are characterized by stability and can adapt to the challenges of the environment to the greatest extent (Láng and Birkás, 2014). The family is a place to live together, and everyone shares and grows up together. Family cohesion can effectively predict individual self-concept (Levenson, 1985). There is a close relationship between self-concept and self-supporting personality (Liu and Jiao, 2013).

In summary, the relationship between family function and individual self-support is logical.

Together, the studies support that individual self-support has an important role with school belonging. Students deal with their affairs, which is conducive to students’ adaptation to the school, to establish school belonging. Previous studies have demonstrated that there is a close relationship between school adaptation and school sense of belonging (Bao and Xu, 2006). Self-support solves the basic problem of individual survival and development (Xia and Huang, 2004). Junior high school students solve their own problems, which is conducive to individual self-development and makes junior high school students feel that they are part of the school. The school works in strict accordance with the schedule. There are fixed arrangements for students when they arrive at school and when they finish a class. The implementation of school rules by students is an important prerequisite for students to integrate into the school. Individuals with high self-support have lower variance in time estimation errors and tend to estimate time intervals more stably (Xia and Chen, 2012). Therefore, self-support junior high school students may better estimate their time and participate in all kinds of school learning. As such, our study provides new evidence for the individual self-support in the association between family function and school belonging.

### The Temporal Path Model

The study proposed a temporal path model to explain how family function leads to school belonging through two parallel mediator variables (interpersonal self-support and individual self-support). Through the study found that the structural equation modeling was supported. In the test of gender difference, we found that the final model showed no gender difference, demonstrating that the final model was stable across genders.

In addition, the mediating effect of interpersonal self-support was stronger than that of individual self-support. Interpersonal self-support was mainly conducive to the solution of interpersonal problems; individual self-support is mainly conducive to individual problem solution solutions (Xia et al., 2011a). Interpersonal independence means being able to engage in basic interpersonal activities by oneself; interpersonal initiative is to take the initiative to interact with others; interpersonal responsibility is to be loyal and trustworthy to others; interpersonal flexibility is to not rigidly adhere to the principles and methods of interpersonal communication, and be able to deal with interpersonal relationships realistically problems, in order to maintain the needs, interests, and face of all parties; open interpersonal is to actively accommodate others (Xia and Huang, 2009). China is a country with collectivist culture. After students go to school, they live and study with others. Junior high school students who are good at getting along with others are more conducive to growing up in a group. Therefore, in the comparison of mediation effect, interpersonal self-support personality can have more influence than individual self-support, mainly related to its attributes. Such analysis is in line with the logic of the study.

### Contributions, Implications, and Limitations

This study demonstrated the parallel mediating role of interpersonal self-support and individual self-support between family function and school belonging. The model and related results expand our understanding of the relationship between family function and school belonging. The perspective of two parallel mediating mechanisms develops and specifies the theoretical views regarding how family function leads to school belonging in the interpersonal self-support and individual self-support.

The study suggests that the effect of interpersonal self-support on school belonging was stronger than that of individual self-support variables. The results suggested that the effect of interpersonal self-support on school belonging in daily school life may be different, and testing and comparing the effect of school belonging-related variables in one model is feasible, which may facilitate our understanding of the occurrence and development of school belonging. It can be seen that paying attention to the construction of family function and promoting the cultivation of junior middle school students’ self-supporting personality plays a key role in developing junior middle school students’ school belonging.

There are some limitations to this research. Firstly, this research design is across-sectional research, so that the research cannot infer the causal relationship between variables. Secondly, the samples selected in this study were some junior high school students in a province of China, and the representativeness of the samples needs to be broadened. Thirdly, the collection of data adopted a self-report method, and the filling of data was vulnerable to the subjective responses of individuals. In future research, the research will be improved according to the above information.

## Data Availability Statement

The original contributions presented in the study are included in the article/supplementary material, further inquiries can be directed to the corresponding author/s.

## Ethics Statement

The studies involving human participants were reviewed and approved by the Academic Ethics Committee of Hunan Normal University. Written informed consent to participate in this study was provided by the participants’ legal guardian/next of kin.

## Author Contributions

ZY and HY: study design and manuscript writing. LP: manuscript writing and data collection. JX: study design and manuscript revising. WX and WH: data collection. All authors contributed to the article and approved the submitted version.

## Conflict of Interest

The authors declare that the research was conducted in the absence of any commercial or financial relationships that could be construed as a potential conflict of interest.

## Publisher’s Note

All claims expressed in this article are solely those of the authors and do not necessarily represent those of their affiliated organizations, or those of the publisher, the editors and the reviewers. Any product that may be evaluated in this article, or claim that may be made by its manufacturer, is not guaranteed or endorsed by the publisher.
